# Supplementary material for: Long Prehensile Protrusions Can Facilitate Cancer Cell Invasion through the Basement Membrane
Source: Cells. 2023 Oct 18;12(20):2474. doi: 10.3390/cells12202474 (PMC10605924; doi:10.3390/cells12202474)
Supplement: Supplementary file 1 [file cells-12-02474-s001.zip › Supplementary materials/cells-2660099-supplementary.pdf]

Supplementary materials

Supplemental videos

Video S1. Protrusion formation and invasion into adjacent collagen by cancer spheroid cells enclosed by a basement membrane. Timelapse, phase-contrast microscopy video movie of an MDA-MB-231BO cancer spheroid encapsulated in a BM spanning 63 hours after polymerization in a 3D collagen hydrogel. Images were acquired every 15 min in 10 μm slices spanning 100 μm. A single frame from this video was published in reference [21].

Video S2. Protrusion extension and pulling on fibrillar collagen matrix. Spinning disk confocal timelapse imaging at the edge of a cancer spheroid of an individual cell (magenta and grayscale, mNeon green-Lifeact) extending a long protrusion and pulling on collagen fibrils (green; Atto565 collagen) over a 10-hour period (~10 hours after spheroid implantation in the hydrogel). Images were acquired every 15 min in 2 μm slices spanning 60 μm. Data shown as a maximum intensity projection of ~20 μm. Nuclei (blue) are labeled with Spy650 DNA.

Supplemental figures

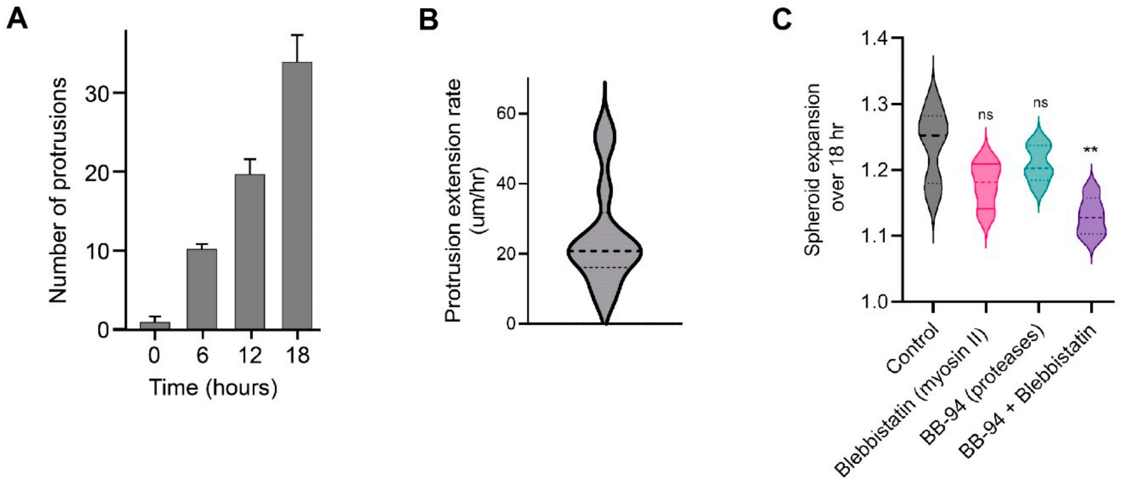

Figure S1. Quantification of the rates of protrusion and spheroid expansion
(**A**) Total number of protrusions per spheroid over time. (**B**) Protrusion rate quantified by measuring individual protrusion lengths and calculating rates of change over time, then shown as a violin plot with the thicker dashed line indicating the median value (21 μm/hr; mean = 25 μm/hr) and the thinner dashed lines indicating quartiles. (**C**) Spheroid diameters measured at time 0 and compared to the diameter of the spheroid at 18 hours, expressed as relative expansion (ratio) and shown as violin plots for the indicated treatments.

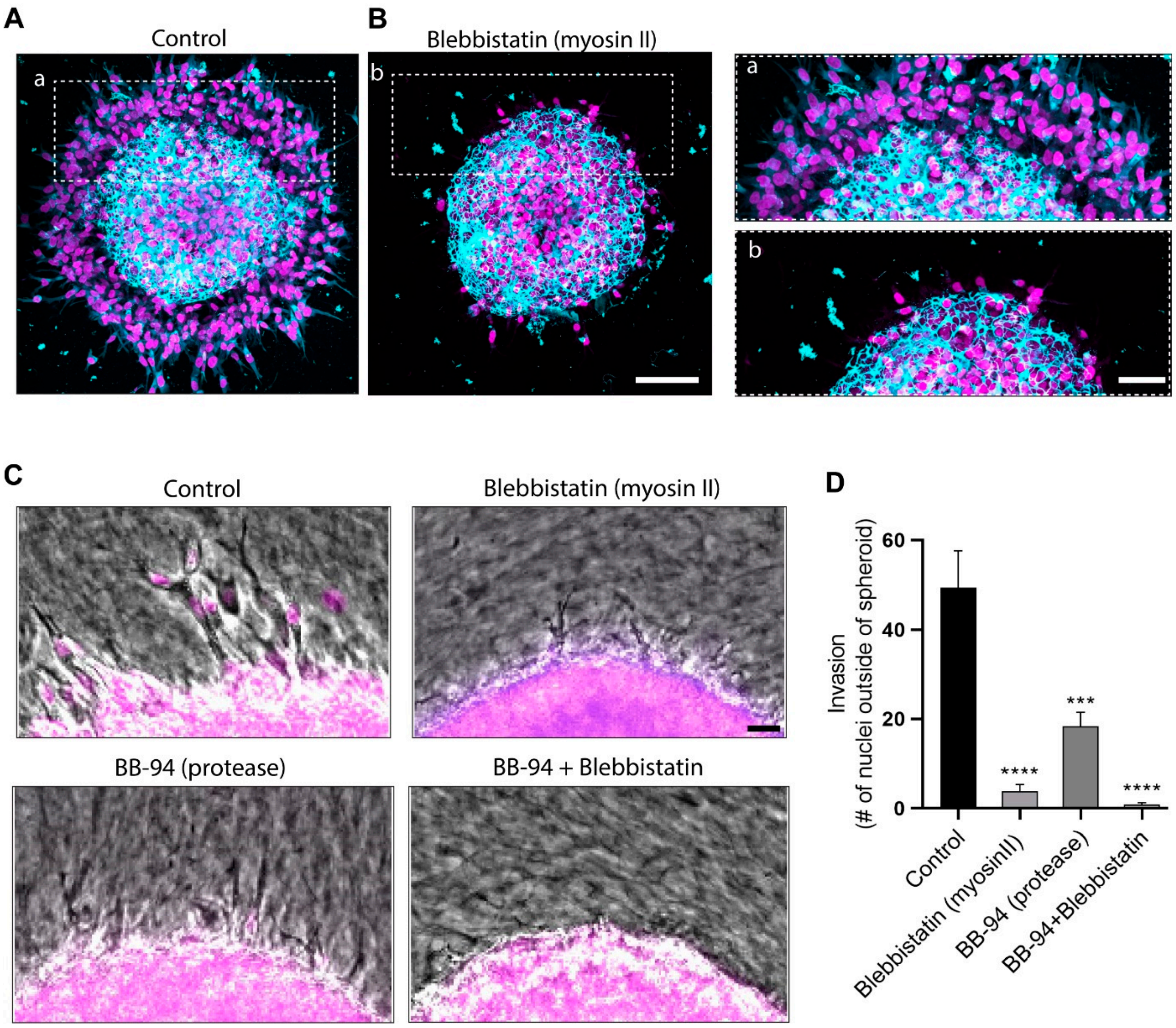

Figure S2. Comparisons of effects of inhibiting contractility and proteases on cancer invasion
Control compared to blebbistatin (myosin II inhibitor), BB-94 (protease inhibitor), and a combination of the two inhibitors. (**A**,**B**) Maximum intensity projection confocal images of the ventral surface of spheroids showing nuclei (magenta) translocated through the basement membrane (collagen IV: cyan) in control compared to blebbistatin-treated representative spheroids at 18 hours with magnified insets (a-b). (**C**) Representative magnified images of spheroids at 18 hours using brightfield microscopy with protease and myosin II inhibitors (magenta fluorescence marks nuclei). (**D**) Quantification of invasion from data such as shown in panel C. Statistical analysis by one-way ANOVA with Dunnett's test in comparison to control: **** $p < 0.0001$, N=3, n=3-7. Mean value ±SEM. Scale bars: (**A**,**B**),100 µm; (a-b insets), 50 µm; (**C**), 20 µm.

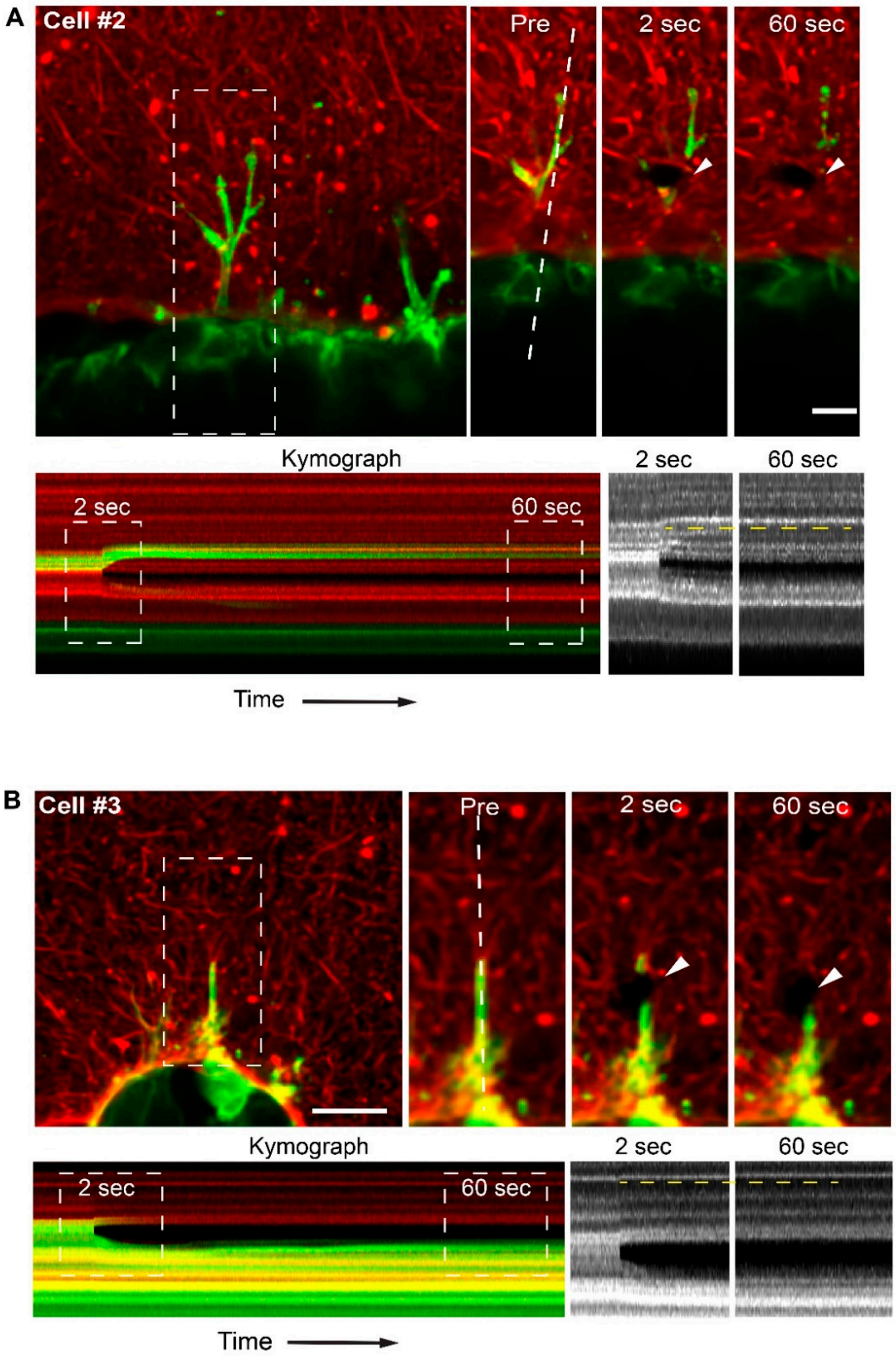

Figure S3. Additional examples of the effects of protrusion ablation on collagen displacement. Data in panels A and B are additional examples demonstrating that severing the cellular protrusion leads to local relaxation of collagen.

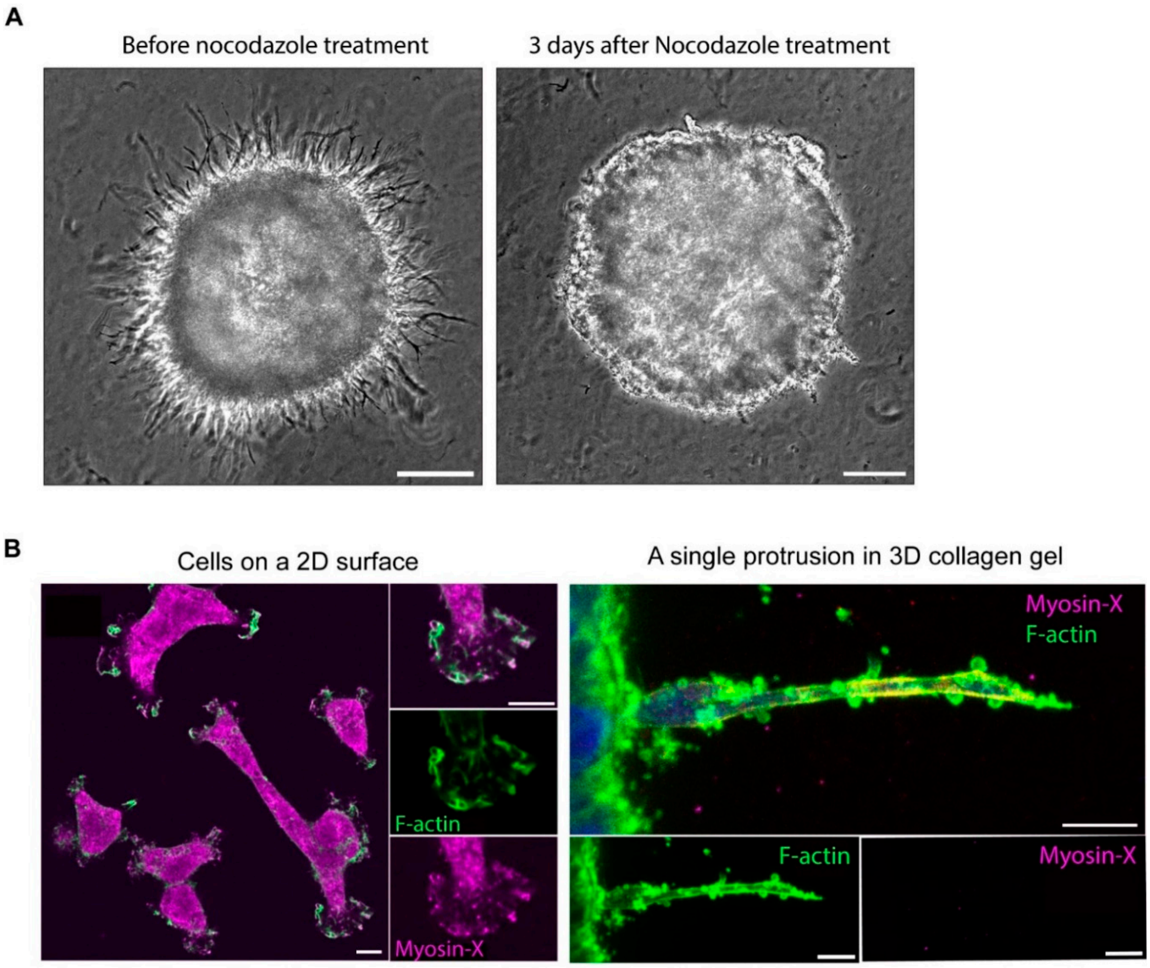

Figure S4. Nocodazole treatment and myosin X staining.

(**A**) To evaluate the role of microtubules in the stabilization of long protrusions, nocodazole was added 24 hours after the spheroids had generated protrusions. (**B**) Although myosin X can be readily detected in the filopodia of cells on a 2-dimensional cell culture surface, it could not be detected in the long invasive protrusions in 3D collagen gels. Scale bars: (**A**) 100 μm; (**B**) 10 μm.

Material	*Source*	*Identifier*
Antibodies		
Goat polyclonal anti-collagen type IV	MilliporeSigma	Cat # AB769
Mouse monoclonal anti $\alpha 2\beta 1$ antibody clone BHA2.1	MilliporeSigma	Cat # MAB1998Z
Rat monoclonal anti β1 antibody	MilliporeSigma	Cat # MABT821
Mouse monoclonal anti-α-tubulin (clone DM1α)	MilliporeSigma	Cat # T6199
Mouse monoclonal anti-Myosin X Antibody (C-1)	Abcam	Cat # sc166720
Rabbit monoclonal Recombinant Anti-non-muscle Myosin IIA antibody	Abcam	Cat # ab236073
Rabbit monoclonal recombinant Anti-non-muscle Myosin IIB antibody	Cell signaling	Cat # ab254472
Mouse monoclonal Anti-PDIA3 antibody	Sigma	Cat # 2446S
Rabbit polyclonal anti-AIF antibody	Abcam	Cat # ab1998
Chemicals, peptides, and proteins		
Blebbistatin	Selleckchem	Cat # S7155
CK-666	Sigma-Aldrich	Cat # 442633-00-3
Nocodazole	Millipore	Cat # M1404-10MG
Cytochalasin-D	Thermo Fisher Sci	Cat # PHZ1063
Latrunculin-A	Thermo Fisher Sci	Cat # L12370
Fascin-G2	Xcess Biosciences	Cat # M60269-10S
Y-27632	Millipore	Cat # 509228
ML141-20	Millipore	Cat # SML0407
Batimastat (BB-94)	TOCRIS	Cat # 296129
Matrigel, Growth Factor Reduced Basement Membrane Matrix	Corning	Cat # 356231
Collagen I	A. Doyle (Yamada laboratory)	Purified in lab
DMSO	Sigma-Aldrich	Cat # D2650
Oxyfluor	Fisher Scientific	Cat # 50-300-2016
Sir-DNA	Cytoskeleton	Cat # CY-SC007
Cell lines		
MDA-MB-231BO	Gift from Kandice Tanner (NCI, NIH), originally generated by [58]	N/A
SCC-9	ATCC	Cat # CRL-1629
Software		
ImageJ (Fiji)	Schindelin et al., 2012	https://fiji.sc
Imaris 9.5.0	Bitplane	https://www.imaris.com

Table S1. Materials and software
